# Microbial life-history strategies and genomic traits between pristine and cropland soils

**DOI:** 10.1128/msystems.00178-25

**Published:** 2025-04-16

**Authors:** Dan He, Zhongmin Dai, Shuxun Cheng, Haojie Shen, Jiahui Lin, Kankan Zhao, Jorge L. Mazza Rodrigues, Yakov Kuzyakov, Jianming Xu

**Affiliations:** 1 Institute of Soil and Water Resources and Environmental Science, College of Environmental and Resource Sciences, Zhejiang University 366101 , Hangzhou, China; 2 Zhejiang Provincial Key Laboratory of Agricultural Resources and Environment, Zhejiang University 12377, Hangzhou, China; 3 The Rural Development Academy at Zhejiang University, Zhejiang University 12377, Hangzhou, China; 4 Department of Land Air, and Water Resources, University of California, Davis, Davis, California, USA; 5 Department of Agricultural Soil Science, University of Göttingen 9375, Göttingen, Germany; 6 Peoples Friendship University of Russia (RUDN University), Moscow, Russia; 7 Institute of Environmental Sciences, Kazan Federal University 64922, Kazan, Russia; University of Massachusetts Amherst, Amherst, Massachusetts, USA

**Keywords:** microbial life-history strategies, genomic traits, land use, fertilization

## Abstract

Microbial life-history strategies [inferred from ribosomal RNA operon (*rrn*) gene copy numbers] and associated genomic traits and metabolism potentials in soil significantly influence ecosystem properties and functions globally. Yet, the differences in microbial strategies and traits between disturbed (cropland) and pristine soils, along with their dominant driving factors, remain underexplored. Our large-scale survey of 153 sites, including 84 croplands and 69 pristine soils, combined with long-term field experiments demonstrates that cropland soils support microbial communities with more candidate r-strategies characterized by higher *rrn* copy numbers and genomic traits conducive to rapid resource utilization. Conversely, pristine soils tend to host communities aligned with more candidate K-strategies marked by high resource use potentials. Elevated nitrogen (N) and phosphorus (P) levels in cropland soils emerge as key factors promoting these candidate r-strategies, overshadowing the influence of organic carbon content, soil structure, or climatic conditions. Results from four long-term field experiments also corroborate that sustained N and P inputs significantly elevate *rrn* copy numbers, favoring these candidate r-strategists. Our findings highlight that land use and fertilization practices critically shape microbial life-history strategies, with nutrient availability being a decisive factor in increasing the r-strategists in cropland soils.

**IMPORTANCE:**

Microbial life-history strategies and genomic traits are key determinants shaping the response of populations to environmental impacts. In this paper, 84 cropland and 69 pristine soil samples were studied, and microorganisms in two ecosystems were categorized into two types of ecological groups using the classical copiotroph–oligotroph dichotomy, promoting a general understanding of the ecological roles of microorganisms. This study is the first to investigate the microbial life-history strategies under different land uses across five climatic zones in China. The results showed that the microbes in cropland soils are more copiotrophic than pristine soils. It also demonstrates that elevated levels of nitrogen and phosphorus in cropland soils are the key factors promoting these r-strategies. This observation emphasizes the critical role of nutrient management in shaping microbial community dynamics and ecosystem functioning and lays the foundation for predicting the response of microbial community composition under resource perturbation.

## INTRODUCTION

Microbial life-history strategies, such as growth rate, resource utilization, and adaptation to environmental conditions, can often be inferred from molecular data, such as the ribosomal RNA operon (*rrn*) gene copy number and genomic traits. The *rrn* copy number is associated with protein synthesis, reflecting microbial candidate r- and K-life strategies (1–3). Copiotrophs associated with r-strategies have a high *rrn* copy number and can rapidly utilize available resources dominating in nutrient-rich environments (2, 4). Oligotrophs, however, with few copies of the *rrn* genes, are more efficient at utilizing complex compounds at the expense of growth rate and dominate in resource-poor environments (5). Microbial traits, such as maximum growth rate, are proportional to the *rrn* copy number, indicating the rapid growth ability of copiotrophs in response to resource addition (6). Oligotrophs containing smaller genome sizes, higher GC contents, and higher abundances of genes encoding metabolic pathways are adapted to carbon and nutrient-poor habitats and have greater survival capacity (7, 8).

Land use changes caused by human activities considerably alter the structure and functions of the soil microbiome. Especially, there are large differences in the microbial community composition, diversity, and functional capabilities between pristine and cropland soils (9–12). The difference in microbial life-history strategies, however, between cropland and pristine soils and the dominant driving factor remains underexplored. The pristine land use types, such as forest and grassland, experience minimal human disturbance and are predominantly influenced by natural factors like climate and edaphic conditions (13). Temperature and precipitation can dictate microbial strategies through the modulation of metabolic rates, enzyme activities, and community composition (14–17). Edaphic factors, such as soil pH, can promote the growth of specific microbial groups by creating districting microhabitats (18).

Cropland soils (e.g., upland, greenhouse, and paddy soils) are subject to intensive agricultural practices, such as fertilization, plowing, and irrigation. Cropland soils receive continuous applications of mineral fertilizers, such as 110–120 Mt yr^−1^ N and 40–45 Mt yr^−1^ P on the globe (19), and supply sufficient N and P for microbial growth. Furthermore, most cropland soils are subjected to plowing and, sometimes, irrigation, which affect bulk density, structure, and oxygen availability (20, 21). These practices alter soil chemistry and structure, providing conditions favorable for microbes that accelerate organic matter decomposition and nutrient cycling (20–22). In contrast, pristine ecosystems (e.g., forests and grasslands) benefit from the continuous accumulation of organic matter (i.e., litter and rhizodeposition) while frequently suffering from N and P limitations (23–25). Despite some site-specific experiments, a comprehensive understanding of differences in microbial strategies between land uses at large scale, particularly inferred by *rrn* copy numbers and associated genomic traits, is lacking (26–29). The strengths of large-scale surveys lie in capturing real-world complexities and providing generalizable insights into how land use shapes microbial strategies across various environmental conditions.

Here, we conducted a large spatial collection of soil samples from 84 cropland ecosystems (i.e., upland, greenhouse, and paddy soils) and 69 pristine soils (forest and grassland without intensive human use) across China. Sample locations are in diverse climatic conditions and have a spatial distribution. We compared the total abundance of 16S rRNA gene, *rrn* copy number at the community level, and microbial genomic traits, such as genome size, GC content, variance of the GC content, and the genes for microbial CNP metabolism between croplands and pristine soils. Our large-scale survey is essential for determining whether the variations in microbial strategies are genuinely attributable to land use changes or if they are obscured by the natural variability within ecosystems, which cannot be revealed by controlled experiments. As large-scale surveys offer less control over experimental variables, we also used four controlled field experiments subjected to long-term agricultural practices to verify the dominant driving factors. The primary objective was to evaluate whether cropland soils have a contrasting microbial life-history strategy compared to pristine soils and what dominant causes lead to the difference. We hypothesized that cropland soils favor the microbial communities with more candidate r-strategies (copiotrophs) due to intensive disturbances, such as excessive nutrient inputs, and pristine soils present higher microbial resource metabolism potentials and resistances to environmental stress (oligotrophs).

## MATERIALS AND METHODS

### Soil sample and characterization

**Fig 1:**
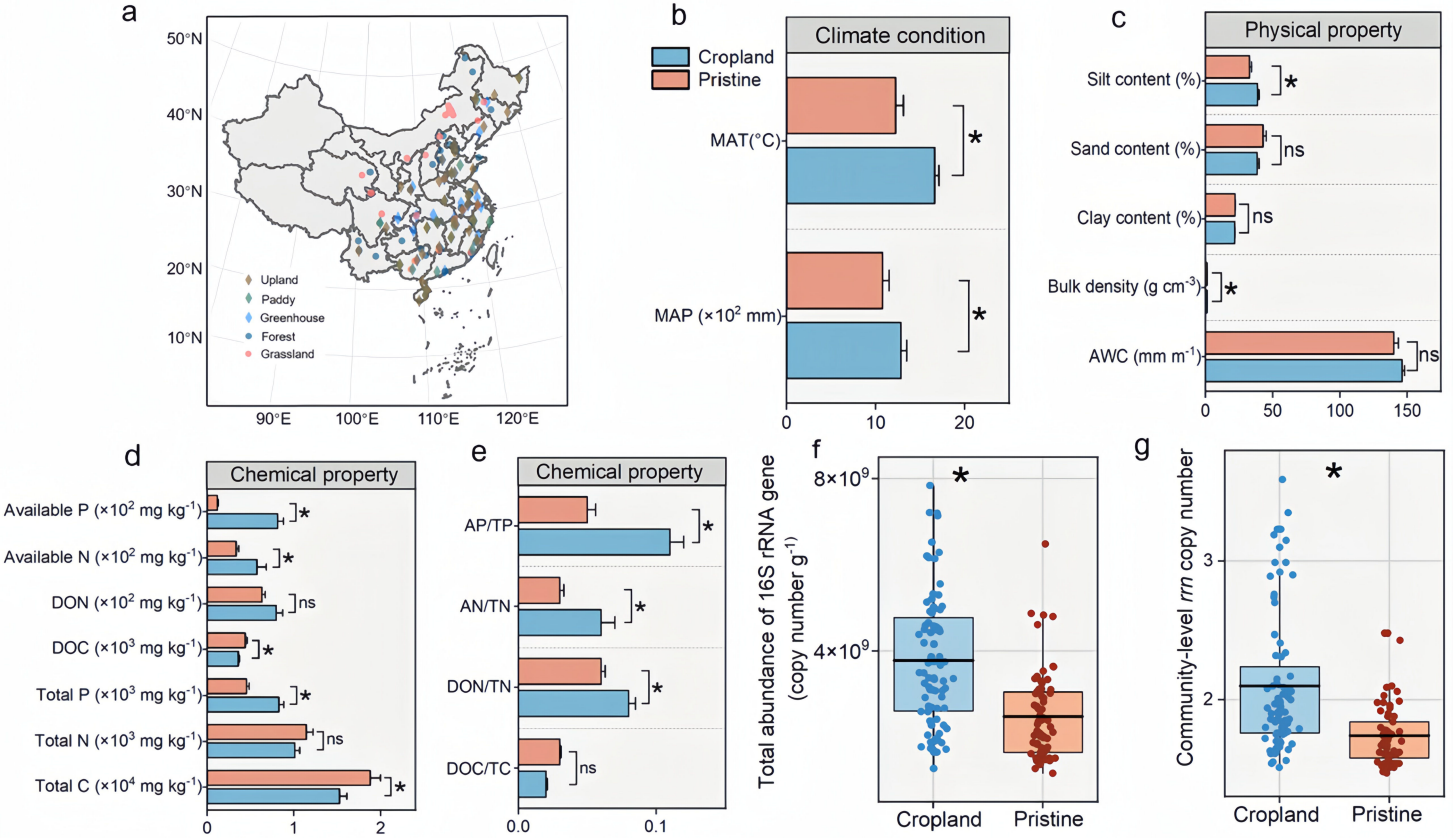
Soil basic properties, total abundance, and community-level *rrn* copy number in cropland and pristine soils. The distribution of 153 sampling sites (a) located in the middle temperate, south temperate, northern subtropical, middle subtropical, and south subtropical zones. Climate condition (b), soil physical property (c), and chemical property (d and e) between cropland and pristine soils. Total abundance of the 16S rRNA gene (f) and average *rrn* copy number (g) between cropland and pristine soils. Box plots indicate the means (horizontal lines), 1st and 3rd quartiles (boxes), and 1.5× interquartile range (whiskers). The differences in the basic properties, total abundance, and community-level *rrn* copy number between cropland and pristine soils were distinguished by the linear mixed-effects models. *P < 0.05, ns: not significant. MAP: mean annual precipitation, MAT: mean annual temperature, AWC: available water capacity, Available P: available phosphorus, Available N: available nitrogen, DON: dissolved organic nitrogen, DOC: dissolved organic carbon, Total P: total phosphorus, Total N: total nitrogen, Total C: total carbon, AP/TP: available phosphorus/total phosphorus, AN/TN: available nitrogen/total nitrogen, DON/TN: dissolved organic nitrogen/total nitrogen, and DOC/TC: dissolved organic carbon/total carbon.

Soil samples in 84 cropland ecosystems (upland, paddy, and greenhouse) and 69 pristine ecosystems (forest and grassland) were collected across China. The greenhouses were the sites with simple structures placed on top of an existing soil habitat. The samples were from the middle temperate, south temperate, northern subtropical, middle subtropical, and south subtropical zones with contrasting climatic conditions (with mean annual precipitation and temperature ranges from 395 to 2486 mm and −2.7 to 27.9°C, respectively) and soil types (Fig. 1a and b). Five samples were taken from the topsoil according to the “S” sampling pattern and mixed into a composite to avoid heterogeneity. Samples were sealed in sterilized plastic bags and transported on ice to the laboratory, with root and stone debris removed. A portion of the samples was stored at −20°C for microbial analysis. Another portion of the samples was air-dried and sieved <2 mm for subsequent chemical analysis. Soil samples were analyzed for chemical properties and DNA extraction synchronously.

The content of soil total carbon (TC) and nitrogen (TN) was measured by an elemental analyzer (Vario EL II, Germany). Total P (TP) was measured using a molybdenum blue colorimetric method after the digestion of soil particles with H_2_SO_4_–HClO_4_. Dissolved organic C (DOC) and dissolved organic N (DON) were determined using a TOC/TN analyzer (multiN/C 3100, Analytik Jena AG, Jena, Germany). Available P (AP) was extracted by 0.5 mol L^−1^ NaHCO_3_ at pH 8.5 and measured according to the Olsen P (30). Soil extractable NH_4_^+^ and NO_3_^−^ were extracted with 1.0 M KCl, and then determined using a continuous flow analytical system (Skalar SAN++ System, Netherlands). The sum of extractable NH_4_^+^ and NO_3_^−^ was called available N (AN). Soil pH was measured in a soil to water suspension of 1:2.5 after shaking for 30 min. The soil available water capacity (AWC%, w/w), bulk density, and texture were collected using the Harmonized World Soil Database v 1.2.

### DNA extraction and sequencing

Soil DNA was extracted using the FastDNA Spin Kit (MP Biomedicals, Solon, OH, USA) according to the manufacturer’s protocol. DNA quality was checked by agarose gel electrophoresis. After library preparation and amplicon sequencing, amplified DNA samples were sequenced on the paired-end platform, NovaSeq 6000 (Illumina, San Diego, CA, USA). The primers of 515F (5′-GTGCCAGCMGCCGCGGTAA-3′) and 907R (5′-CCGTCAATTCCTTTGAGTTT-3′) targeted the V4 region of the bacterial 16S rRNA gene. The raw data quality was meticulously controlled using Fastp (https://github.com/OpenGene/fastp), employing a sliding window configuration (-W 4, -M 20), and primers were removed through cutadapt (https://github.com/marcelm/cutadapt/).

On average, we retained 65,866 high-quality reads for bacteria. Subsequently, in the QIIME 2 pipeline, we merged the paired-end reads and generated the feature table using the DADA2 denoise-paired plugin, with the trimming of chimeric reads following the method (31). To assign taxonomy to these sequences, we utilized the q2-feature classifier plugin (https://github.com/qiime2/q2-feature-classifier), employing the Silva 138 database with a default confidence threshold of 0.7. In total, 98,805 amplicon sequence variants (ASVs) were generated for bacteria and fungi. Bacterial alpha diversity of the Shannon and Chao 1 indices was obtained by rarefying all sequences at a minimum number of sequences (i.e., 33,018). Microbial beta diversity was performed using the R package “phyloseq” based on the weighted unifrac distance matrix and revealed by the nonmetric multidimensional scaling plots (NMDS). Raw sequencing data were deposited in the Sequence Read Archive (SRA) with accession number PRJNA1084892.

The *rrn* copy numbers for individual ASVs were estimated based on the closest strain match in the *rrn*DB database27 (version 5.4, https://rrndb.umms.med.umich.edu/) (32). The community-level average *rrn* copy number, which represents the overall bacterial life-history strategies in one soil sample, was calculated as the mean of the estimated *rrn* copy number after the relative abundance for each ASV was weighted. The estimated *rrn* copy numbers are widely used to indicate the microbial r- and K- life-history strategies (4, 33). The equation of the community-level average *rrn* copy number was as follows:

Community-level average *rrn* copy number = $\sum_{i=1}^{N}Ri/\sum_{i=1}^{N}\frac{Ri}{Wi}$,

where *N* is the number of ASVs in a soil sample; *Ri* is the sequence abundance of *ASV*_*i*_; and *Wi* is the estimated *rrn* copy number of *ASV*_*i*_.

### Metagenomics

To identify the microbial genomic traits associated with the predicted maximum growth rate, predicted minimum generation rate, genome size, GC content, and variance of the GC content, we conducted metagenomic sequencing on all DNA samples. Paired-end sequencing was performed using the NovaSeq 6000 (Illumina, Inc.). The quality of raw reads was controlled by using fastp. The filtered reads were assembled into contigs using Megahit, utilizing a de Bruijn graph approach, with minimum and maximum k-mer sizes set at 21 and 121 (34), respectively. Contigs with a length greater than 500 base pairs (bp) were retained for further analysis. Next, Prodigal was used to identify the open reading frames in contigs and predict the protein-coding gene (35). Kyoto Encyclopedia of Genes and Genomes (KEGG) functional annotations were performed using DIAMOND against the KEGG database with an e-value cutoff of <1 × 10⁻⁵. Relative gene abundances were calculated as the proportion of annotated reads for each functional category. The number of genomes was estimated as described by Pereira-Flores et al. (36), and the average genome size was determined by dividing the total number of base pairs by the number of genomes. Furthermore, the codon usage bias in ribosomal genes and the predicted minimum generation time (h) were computed following the methodology outlined in Vieira-Silva and Rocha (6). The predicted maximum growth rate was derived as the reciprocal of the predicted minimum generation time (h^−1^) (37). The calculation of the GC content and the variance of the GC contents in the quality-filtered reads were performed according to the procedures outlined in Barberán et al. (38). These traits are closely linked to microbial life-history strategies (3, 39) (Table S1).

### High-throughput qPCR-based chip

In addition, the total absolute abundance of the 16S rRNA gene was measured by qPCR using the same primers for 16S sequencing. In brief, the amplification cycling condition was as follows: (i) initial denaturation at 95°C for 5 min; (ii) 40 cycles of denaturation at 95°C for 15 s; (iii) annealing at 60°C for 30 s; and (iv) extension at 72°C for 30 s. The absolute abundance of 66 genes involved in C, N, and P metabolism (Table S3) was quantified using a high-throughput qPCR-based chip (40). This chip was performed on the SmartChip Real-time PCR System (WaferGen Biosystems, Fremont, USA) following the manufacturer’s instructions. The qPCR amplification protocol had the following steps: (i) an initial denaturation at 95°C for 10 min; (ii) 40 cycles of denaturation at 95°C for 30 s; (iii) annealing at 58°C for 30 s; and (iv) extension at 72°C for 30 s. Each DNA sample was amplified in triplicate. Samples were excluded from further analyses if their amplification efficiencies were <1.8 or >2.2, negative control-amplified, or the threshold cycle (CT) value > 31. The relative abundance of specific functional genes was calculated by dividing their absolute abundance by the absolute abundance of the 16S rRNA gene. The cumulative relative abundances of all functional genes involved in carbon (C), nitrogen (N), and phosphorus (P) cycling, as quantified by qPCR, are presented in Fig. 3a. The relative abundances of 48 genes associated with well-established pathways for carbon hydrolysis, inorganic nitrogen transformation, and phosphorus solubilization, as identified in previous studies (41–43), are shown in Fig. 3b through d.

### Data analysis

The results of the total abundance of the 16S rRNA gene, average *rrn* copy number, alpha-diversity, and beta-diversity were spatially interpolated using the inverse distance weighted spatial interpolation in ArcGIS 10.8 (44). The microbial alpha diversity revealed by Shannon and Chao 1 was obtained before rarefying all sequences at a minimum number of sequences per sample. Microbial beta diversity revealed by NMDS1 and NMDS2 was performed using the R package “phyloseq” based on the weighted unifrac distance matrix (45). The differences in the total abundance of the 16S rRNA gene, average *rrn* copy number, genomic traits, soil nutrient content, microbial taxonomic abundances, and functional gene abundances between cropland and pristine soils were performed by the linear mixed-effects models using lme4 and lmerTest packages (46). In the model, the ecosystem type was used as a fixed effect, and the site was treated as a random effect. The “DESeq2” identified the responsive bacterial genera with a log2-fold change in relative abundance >1 and an adjusted *P* < 0.05 using cropland soils as compared with pristine soils (47). The relationships between *rrn* copy numbers and soil nutrients/functional gene abundances were analyzed by the Spearman correlation.

Structural equation modeling (SEM) was employed to quantify the contributions of soil properties and environmental factors to the average *rrn* copy number and the total abundance of the 16S rRNA gene. We hypothesized that C-, N-, and P-related parameters in the soil directly and indirectly affect (by altering the soil microbial community) the microbial *rrn* copy numbers. To consolidate the soil parameters, we conducted principal component analyses (PCA) for total soil C and available C, with the first principal component (PC1) representing the “C parameter.” The “N parameter” and “P parameter” were obtained in the same approach. Likewise, we created a climatic parameter by extracting PC1 from PCA results for mean annual precipitation and mean annual temperature. For constructing the SEM, we utilized AMOS 24.0 (SPSS, Chicago, IL, USA) with maximum likelihood estimation to fit the covariance matrix within the model. The model assessment criteria included the *χ* test (*P* > 0.05) and the root mean square error of approximation (RMSEA < 0.05) in line with the methodology outlined by Grace and Keeley (48).

All predictors and response variables were normalized with Z-scores to explain parameters on comparable scales. Using the “MuMIn” package (49), we generated a set of models containing all possible combinations of initial predictors. Then, the models were ranked according to the Akaike Information Criterion (AIC) of the maximum likelihood fit in r. Subsequently, the optimal model based on the minimum value of the AIC (function dredge) was determined by Zhang et al. (50). Then, the relative effects of the parameter estimates for each predictor compared to the effects of all parameter estimates were calculated (function rdacca.hp). Based on the final optimal model, six variables (NMDS2, Shannon, N + P parameter, AWC, C parameter, bulk density) with a high explanatory degree are retained for the average *rrn* copy number and five variables (NMDS2, Shannon, N + P parameter, C parameter, pH) for the total abundance of the 16S rRNA gene.

### Agro-ecosystems with long-term inorganic fertilization

We collected soil samples at four experimental sites that had been treated with NP fertilizers. These sites encompassed four distinct soil types: red soil (116°20′E, 28°15′N), brown soil (123°34′E, 41°49′N), black soil (126°35′E, 45°40′N), and brick red soil (113°26′E, 23°23′N). These soils have been receiving NP fertilizers (+NP) for 31, 30, 39, and 7 years, respectively, with three replicate plots, and the soils having no fertilizers were included as the controls (−NP). The annual application rates of N and P in red soil were urea (120 kg N ha^−1^) and calcium magnesium phosphate (26 kg P ha^−1^). The annual application rates of N and P in black soil were urea (375 kg N ha^−1^) and calcium superphosphate + diammonium phosphate (131 kg P ha^−1^). The annual application rates of N and P in brown soil were urea (150 kg N ha^−1^) and ammonium dihydrogen phosphate (59 kg P ha^−1^). The annual application rates of N and P in brick red soil were urea (720 kg N ha^−1^) and ammonium dihydrogen phosphate (241 kg P ha^−1^). The average *rrn* copy numbers in the plots with and without NP fertilizers were measured as previously described.

## RESULTS

### Distribution of microbial life-history strategies

We constructed spatial maps of the total abundance of the 16S rRNA gene, average *rrn* copy number, α-diversity, and β-diversity of soil microbial communities for whole sampling regions across China (4, Fig. S1). Western China showed the highest total abundance of the 16S rRNA gene, average *rrn* copy number, and lower Shannon, Chao1, and NMDS1. There was a higher total abundance of the 16S rRNA gene in central and eastern regions, while the northern and southern regions had the opposite trend (Fig. S1). The average *rrn* copy number showed opposite spatial patterns to α- and β-diversities (Fig. S1).

### Soil basic properties, microbial abundance, and *rrn* copy numbers

The nutrient availability in cropland soils was higher than that in pristine soils (Fig. 1). Available water capacity, clay content, and sand content were similar (*P* > 0.05) between pristine and cropland soils, while soil bulk density and silt content were 0.03 g cm^−3^ and 5.9% higher (*P* < 0.05) in pristine soils, respectively (Fig. 1c). The available P and available N contents in cropland soils were 133 and 373 mg kg^−1^ higher (*P* < 0.05) than in pristine ecosystems, respectively (Fig. 1d). The dissolved organic carbon (DOC) had the opposite trend (*P* < 0.05), and the dissolved nitrogen (DON) remained at a similar level. The total C content was higher by 0.35% (*P* < 0.05) in pristine soils, while the total P content was 0.04% higher (*P* < 0.05) in cropland soils. The absence of differences in total N between cropland and pristine soils was observed (Fig. 1d). The contents of available P and available N (NH_4_^+^ + NO_3_^−^) and the ratios of AP/TP, AN/TN, and DON/TN in cropland soils were (*P* < 0.05) higher than pristine soils. However, the DOC/TC ratio was similar between cropland and pristine soils (Fig. 1e).

The total abundance of the 16S rRNA gene in the cropland soils was 1.3 × 10^9^ (copy number g^−1^) higher (*P* < 0.05) than that in pristine soils (Fig. 1f). Consistently, the average *rrn* copy number was 0.36 (i.e., 20%) higher in cropland soils than that in pristine soils (Fig. 1g). However, the levels of the average *rrn* copy number were similar (*P* > 0.05) among four climate zones, except the middle subtropical zone (Fig. S2). The total abundance of the 16S rRNA gene increased with the increasing average *rrn* copy number across all samples, regardless of the ecosystem type (Fig. S3).

### Microbial genomic traits and oligotrophy/copiotrophy abundance

**Fig 2:**
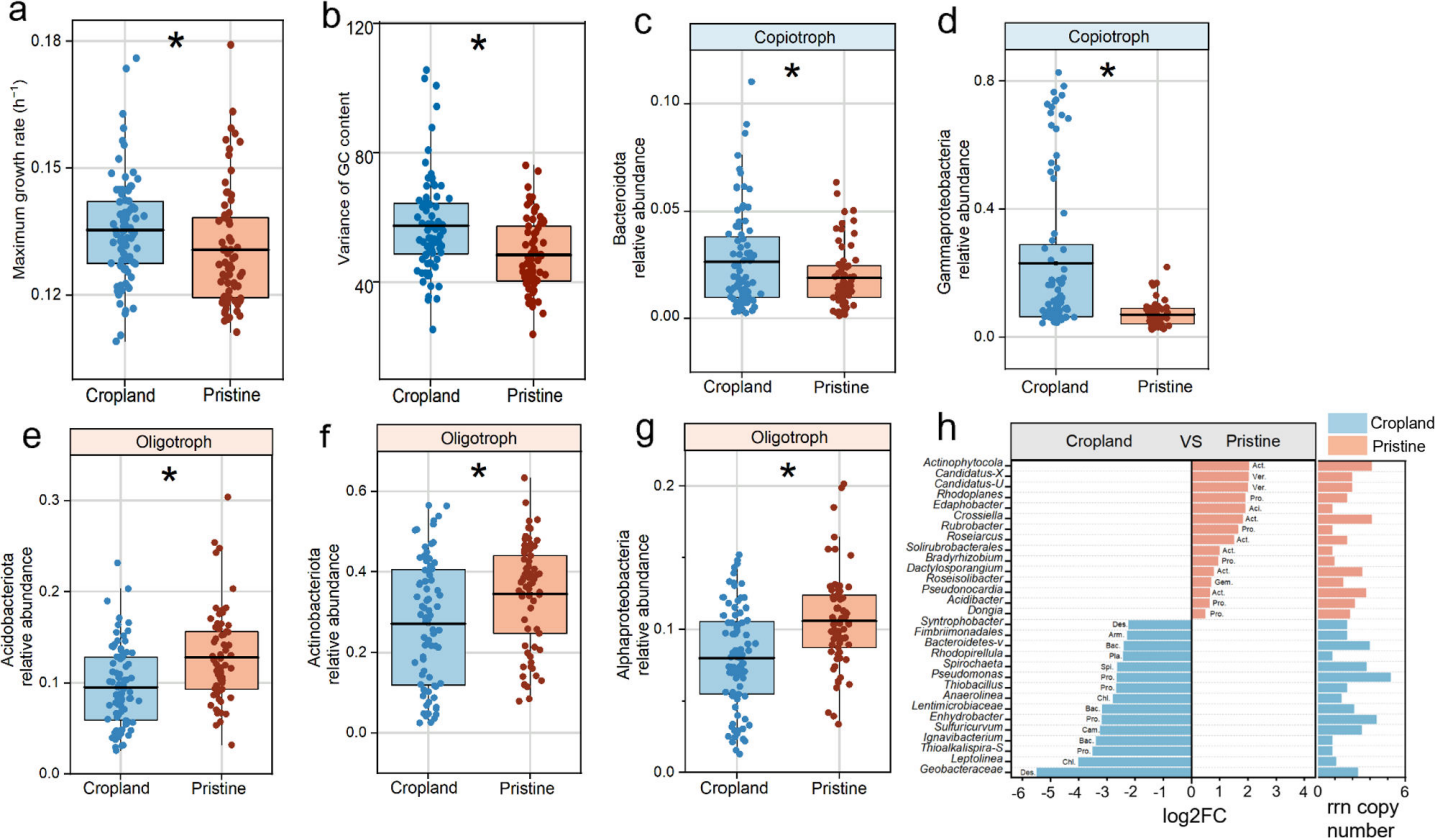
Predicted maximum growth rate, variance of the GC content, and abundances of dominant copiotrophs and oligotrophs between cropland and pristine soils. Predicted maximum growth rate (a) and variance of the GC content (b) between cropland and pristine soils. Typical copiotrophs of *Bacteroidetes* (c) and *Gammaproteobacteria* (d) and oligotrophs of *Acidobacteria* (e), *Actinobacteria* (f), and *Alpha-proteobacteria* (g) between cropland and pristine soils. According to DESeq2 analyses (*P* < 0.05 after FDR correction), the genera were only shown to be significantly more abundant in cropland (blue) or pristine (red) soils (h). The right part of (h) represents the *rrn* copy number corresponding to each genus filtered by DESeq2. “*Candidatus-X*,” “*Candidatus-U*,” “*Bacteroidetes-v*,” and “*Thioalkalispira-S*” are the abbreviations for *Candidatus-Xiphinematobacter*, *Candidatus-Udaeobacter*, *Bacteroidetes-vadinHA17*, and *Thioalkalispira-Sulfurivermis*, respectively. Box plots indicate the means (horizontal lines), 1st and 3rd quartiles (boxes), and 1.5× interquartile range (whiskers). The differences between cropland and pristine soils were distinguished by the linear mixed-effects models. *P < 0.05, ns: not significant.

The cropland soils favored the microbial communities with traits associated with more candidate r-strategies compared to pristine soils (Fig. 2; Fig. S4). The predicted maximum growth rate was 0.004 (h^−1^) higher (*P* < 0.05) for microbial communities in cropland soils than those in pristine soils (Fig. 2a). Similarly, the predicted minimum generation time was 0.29 (h) lower (*P* < 0.05) in cropland soils (Fig. S4a). The variance of the genomic GC content in pristine soils was 9.1 lower (*P* < 0.05) than that in cropland soils (Fig. 2b), while the GC content, genome size, and codon usage bias were similar (*P* > 0.05) between the two ecosystems (Fig. S4b through d).

Microorganisms in cropland soils had greater relative abundances of genes associated with folding, sorting and degradation, signaling molecules and interactions, transport and catabolism, energy metabolism, glycan biosynthesis and metabolism, and metabolism of cofactors and vitamins (*P* < 0.05) (Table 1). In contrast, microorganisms in pristine soils had a higher (*P* < 0.05) relative abundance of genes related to amino acid metabolism, metabolism of other amino acids, xenobiotic biodegradation and metabolism, membrane transport, cell growth and death, and cellular community prokaryotes (Table 1).

**TABLE 1:** Relative abundances of KEGG categories in the cropland and pristine soils (mean ± standard error)^*a*^

KEGG category	Mean ± SE		*P* value
	Cropland soil	Pristine soil	
Energy metabolism	0.085 ± 0.0004	0.083 ± 0.0004	**<0.05^*b*^**
Biosynthesis of other secondary metabolites	0.0244 ± 0.0001	0.0244 ± 0.0001	>0.05
Glycan biosynthesis and metabolism	0.029 ± 0.0002	0.028 ± 0.0002	**<0.05**
Lipid metabolism	0.0385 ± 0.0001	0.0384 ± 0.0002	>0.05
Metabolism of cofactors and vitamins	0.067 ± 0.0003	0.066 ± 0.0003	**<0.05**
Metabolism of amino acids	0.113 ± 0.0005	0.114 ± 0.0004	**<0.05**
Metabolism of other amino acids	0.031 ± 0.0001	0.032 ± 0.00007	**<0.05**
Metabolism of terpenoids and polyketides	0.0192 ± 0.0001	0.0189 ± 0.0001	>0.05
Nucleotide metabolism	0.0394 ± 0.0003	0.0393 ± 0.0003	>0.05
Xenobiotics biodegradation and metabolism	0.034 ± 0.0003	0.035 ± 0.0003	**<0.05**
Carbohydrate metabolism	0.1196 ± 0.0003	0.1192 ± 0.0003	>0.05
Folding, sorting, and degradation	0.027 ± 0.0001	0.026 ± 0.0001	**<0.05**
Replication and repair	0.0295 ± 0.0002	0.0296 ± 0.0002	>0.05
Transcription	0.0051 ± 0.00006	0.0052 ± 0.00007	>0.05
Translation	0.0505 ± 0.0005	0.0493 ± 0.0005	>0.05
Membrane transport	0.051 ± 0.00026	0.053 ± 0.00029	**<0.05**
Signal transduction	0.0474 ± 0.0003	0.0465 ± 0.0003	>0.05
Signaling molecules and interaction	0.0002 ± 0.00001	0.0001 ± 0.00001	**<0.05**
Cell growth and death	0.0185 ± 0.0001	0.0192 ± 0.0001	**<0.05**
Cell motility	0.0107 ± 0.0002	0.0107 ± 0.0002	>0.05
Cellular community—prokaryotes	0.048 ± 0.0005	0.05 ± 0.0005	**<0.05**
Transport and catabolism	0.0068 ± 0.00006	0.0064 ± 0.00006	**<0.05**

^*a*^ Differences in the relative abundances in cropland and pristine soils were investigated by linear mixed-effects models. Four categories of metabolism, genetic information processing, environmental information processing and cellular processes are shown.

^*b*^ Bold values represent significant differences (*P* < 0.05).

### Oligotroph and copiotroph abundance

The cropland soils favored more candidate copiotrophs than oligotrophs compared to pristine soils (Fig. 2). At the phylum level, the relative abundance of *Bacteroidetes* (generally close to copiotrophs) in cropland soils was 1% higher than those in pristine soils (Fig. 2c), while that of *Firmicutes* was at the same level in cropland and pristine soils (Fig. S4e). Among the *Proteobacteria*, the abundance of *Gammaproteobacteria* was higher in cropland soils (Fig. 2d). The relative abundances of *Acidobacteria* and *Actinobacteria* (generally close to oligotrophs) in cropland soils were 3 and 7% lower than those in pristine soils, respectively (Fig. 2e and f), and the *Alphaproteobacteria* (Fig. 2g) (oligotrophs) had a higher abundance in pristine soils.

Especially, the *Bacteroidetes-vadinHA17* (Bacteroidota), *Pseudomonas* (Proteobacteria), and *Enhydrobacter* (Proteobacteria) with the individual *rrn* copy number of 3.5, 5, and 4, respectively, were identified as the genera that had higher abundances in cropland soils (Fig. 2h). By contrast, the *Edaphobacter* (Acidobacteria), *Solirubrobacterales* (Actinobacteria), and *Roseiarcus* (Actinobacteria) with the individual *rrn* copy numbers of 1, 1, and 2, respectively, were identified to have higher abundances in pristine soils (Fig. 2h).

### Microbial metabolism potentials

**Fig 3:**
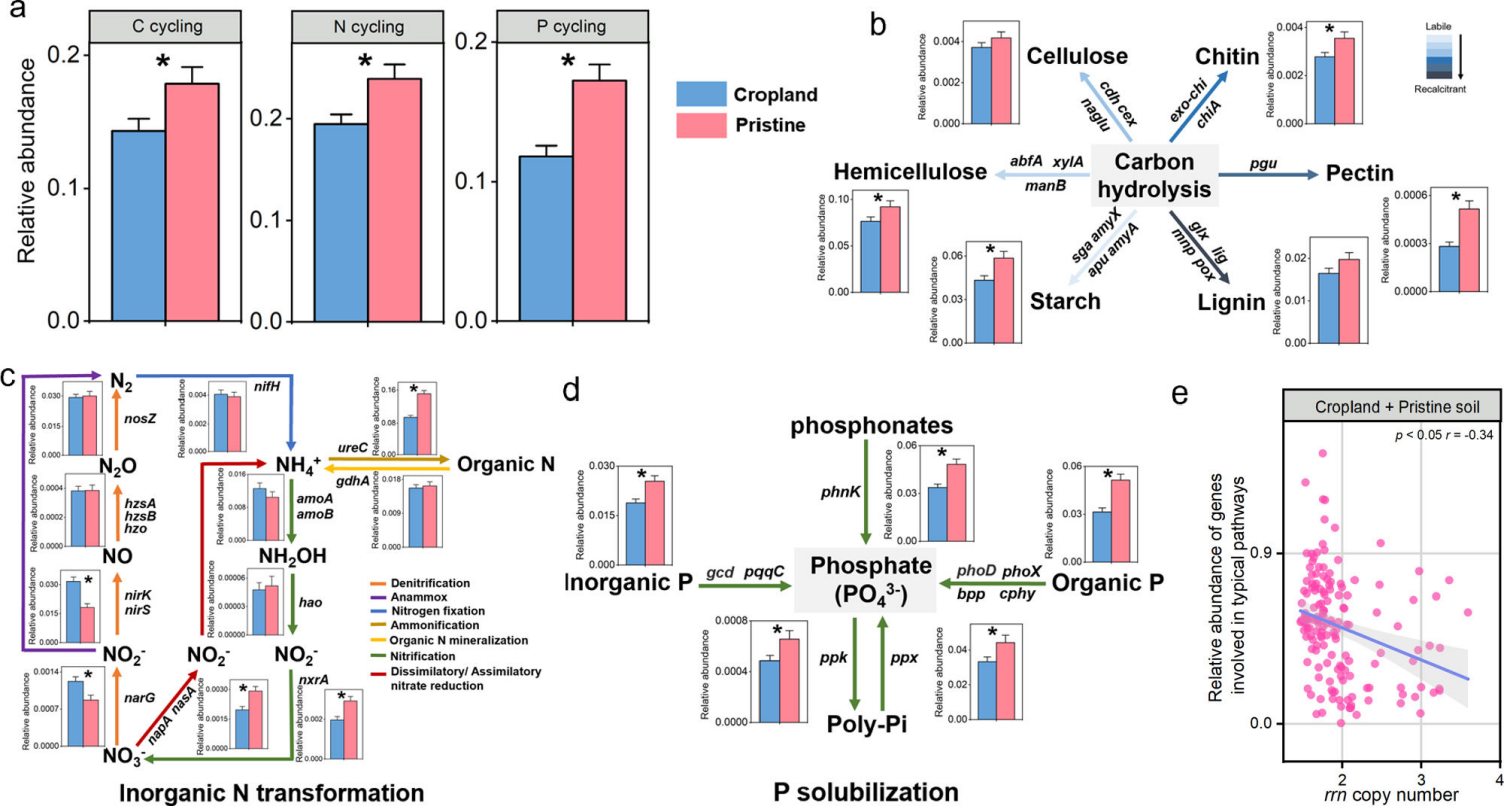
Microbial gene abundances for C, N, and P cycling between cropland and pristine soils. Sum of relative abundances of all the genes involved in C, N, and P cycling measured by the qPCR (a) in cropland and pristine soils. The relative abundances of microbial genes participated in the typical pathways of carbon hydrolysis (b), inorganic N transformation (c), and P solubilization (d). Spearman correlation between the *rrn* copy number (e) and the relative abundances of C, N, and P genes involved in the typical pathways. The differences in the microbial gene abundances between cropland and pristine soils were performed by the linear mixed-effects models. Blue for cropland soils and red for pristine soils. Error bars indicate the standard error. *P < 0.05. Genes involved in carbon hydrolysis (*amyX*, *apu*, *amyA*, *sga*, *abfA*, *manB*, *xylA*, *cdh*, *cex*, *naglu*, *chiA*, *exo-chi*, *pgu*, *glx*, *lig*, *mnp*, *pox*), inorganic N transformation (*napA*, *gdhA*, *nasA*, *nirK1*, *nirK3*, *nirS2*, *nosZ1*, *narG*, *nirK2*, *nirS1*, *nirS3*, *nosZ2*, *nifH*, *amoB*, *amoA1*, *nxrA*, *hao*, *amoA2*, *ureC*, *hzo*, *hzsA*, *hzsB*), and P solubilization (*phoD*, *cphy*, *bpp*, *phoX*, *phnK*, *pqqC*, *gcd*, *ppk*, *ppx*). All the gene abundances were measured by high-throughput, qPCR-based chip. The relative abundance of each gene was calculated by dividing their absolute abundance by the absolute abundance of the 16S rRNA gene.

The pristine soils favored the microbial communities with higher metabolism potentials compared to cropland soils (Fig. 3). The abundances of genes involved in microbial metabolisms of C metabolism, organic N mineralization, and organic P mineralization were higher (*P* < 0.05) in pristine than in cropland soils (Fig. 3a). Specifically, genes for the C metabolism involved in starch (*sga*, *amyX*, *apu*, and *amyA*), hemicellulose (*abfA*, *xylA*, and *manB*), pectin (*pgu*), and chitin (*exo-chi* and *chiA*) decomposition had lower abundance (*P* < 0.05) in the cropland soil compared to pristine soils (Fig. 3b). N metabolism genes involved in dissimilatory/assimilatory nitrate reduction (*nasA* and *napA*) and ammonification (*ureC*) were higher (*P* < 0.05) in pristine soils than in cropland soils. However, the genes responsible for denitrification (*narG*, *nirS*, and *nirK*) had higher (*P* < 0.05) abundance in cropland soils (Fig. 3c). Regarding the P metabolism, the abundances of genes encoding inorganic P solubilization (*gcd* and *pqqC*), organic P mineralization (*phoD*, *phoX*, *bpp*, *cphy*, and *phnK*), inorganic P biosynthesis (*ppk*), and inorganic P hydrolysis (*ppx*) were significantly higher (*P* < 0.05) in pristine soils in comparison to those observed in cropland soils (Fig. 3d). Gene abundances for microbial total metabolisms had a negative correlation with the average *rrn* copy number (*r* = −0.34, *P* < 0.05) and total abundance of the 16S rRNA gene (*r* = −0.41, *P* < 0.05) (Fig. 3e).

### Dominant driving factors

**Fig 4:**
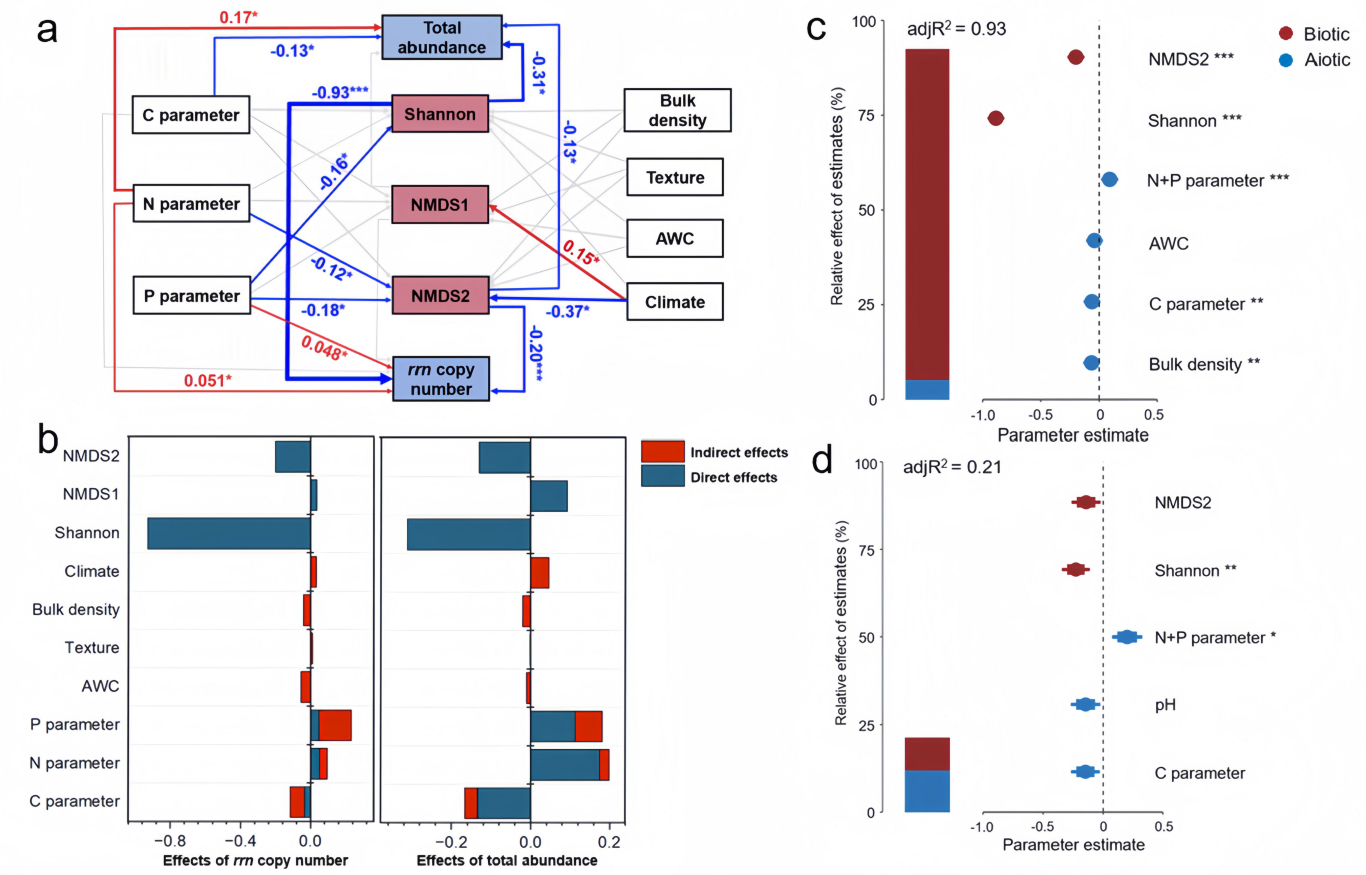
Contributions of climatic conditions and soil biotic and abiotic properties to microbial life-history strategies. SEM model of the contributions of nutrients, soil structure parameters, microbial diversity, and climate to the microbial average *rrn* copy number and total abundance of the 16S rRNA gene (a). Direct or indirect effects of environmental variables on the microbial average *rrn* copy number and total abundance of the 16S rRNA gene (b). Relative effects of multiple predictors of the average *rrn* copy number (c) and total abundance of the 16S rRNA gene (d). It shows the relative importance of each predictor expressed as the percentage of explained variance, as well as the average parameter estimates (standardized regression coefficients) of the model’s predictors and their 95% CIs. In SEM, red and blue arrows represent significant positive and negative correlations (*P* < 0.05) between variables, respectively, while arrows with insignificant relationships are connected by light gray lines. Adjacent values near the arrows indicate path coefficients. *P < 0.05; **P < 0.01; ***P < 0.001. AWC: available water capacity.

Soil N and P (*P* < 0.05) rather than organic C content, soil structure, and climate influenced the average *rrn* copy number (Fig. 4a and b). The structural equation model showed that soil N + P nutrient content had larger total effects on the average *rrn* copy number (32.5%) and the total abundance of the 16S rRNA gene (38%) than soil organic C, climate, and soil physical properties. Specifically, the soil C parameter had no effects (*P* > 0.05) on the average *rrn* copy number but contributed (*P* < 0.05) to the total abundance of the 16S rRNA gene. The soil N parameter directly increased (*P* < 0.05) contribution to the average *rrn* copy number and the total abundance of the 16S rRNA gene. The N parameter affected the microbial community composition (i.e., NMDS2) and further contributed to the average *rrn* copy number. The soil P parameter also directly contributed (*P* < 0.05) to the average *rrn* copy number. Furthermore, the P parameter affected microbial community composition (i.e. NMDS2) and alpha diversity (i.e. Shannon), further contributing to the average *rrn* copy number and total abundance of 16S rRNA gene. Soil bulk density, texture, and moisture did not contribute (*P* > 0.05) to microbial life-history strategies, while climate affected the average *rrn* copy number by affecting the microbial community composition (i.e., NMDS2).

Multiple regression models indicated that the soil N + P parameter, C parameter, and bulk density were the important predictors (*P* < 0.05) of the *rrn* copy number (Fig. 4c). While the soil N + P parameter was the predictor (*P* < 0.05) of the total abundance of the 16S rRNA gene (Fig. 4d). In the best model, the soil N + P parameter was the predictor (*P* < 0.05) of the average *rrn* copy number and total abundance of the 16S rRNA gene (Fig. 4c and d).

### NP fertilization effects on microbial strategies

**Fig 5:**
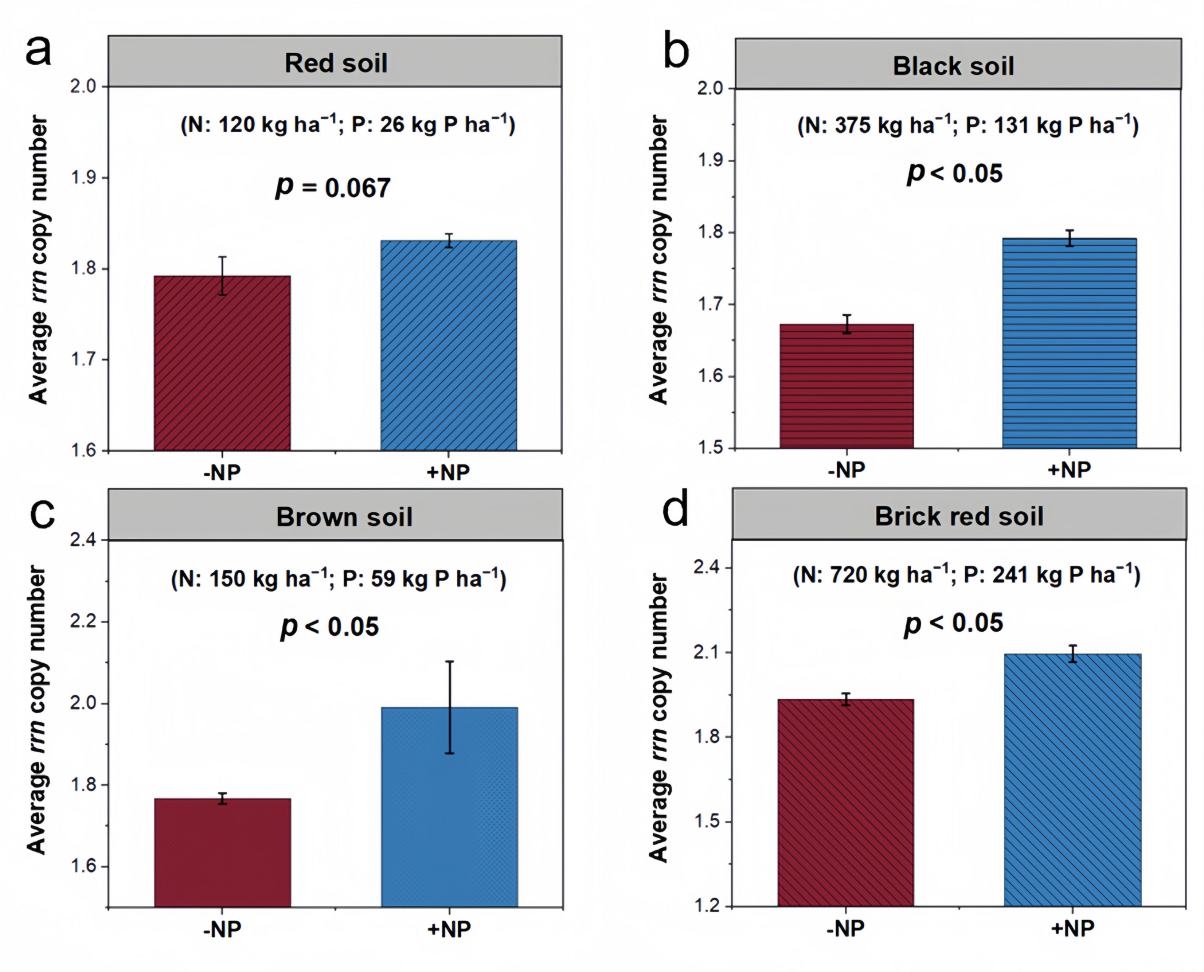
Effects of long-term NP fertilizations on soil microbial life-history strategies. Community-level average *rrn* copy numbers under control and NP fertilization treatments at four long-term field sites with red soil (a), black soil (b), brown soil (c), and brick red soil (d). Differences in the *rrn* copy number at the community level between the NP fertilized and unfertilized soils were analyzed using a linear mixed-effects model.

The average *rrn* copy number was responsive to the long-term NP fertilization across four different agroecosystems receiving different amounts and durations of NP fertilization in comparison to a control soil at the same site, which remained unfertilized (red soil = lowest NP fertilization for 31 years, brown soil = intermediate NP for 30 years, black soil = high NP for 39 years, and brick red soil = highest NP fertilization for 7 years, details in methods). With the red soil receiving relatively less NP fertilizers compared to the other three soils, long-term NP fertilization had no effects on the average *rrn* copy number (*P* = 0.067) (Fig. 5a). However, long-term NP fertilization increased the average *rrn* copy numbers by 0.12, 0.22, and 0.16 in black (*P* < 0.05), brown (*P* < 0.05), and brick red soils (*P* < 0.05), respectively (Fig. 5b through d).

## DISCUSSION

Microbial life-history strategies play a pivotal role in regulating soil nutrient cycling and ecosystem functioning (3, 51, 52). Microbial communities with a high number of *rrn* copies have, in general, more copiotrophs, which have fast growth rates (Table S1), while the communities with fewer *rrn* copy numbers have more oligotrophs (1, 4, 33). Recent studies have confirmed that using the average *rrn* copy number and genomic traits predicted from bioinformatic analyses of amplicon sequencing and metagenomics data is an effective approach for inferring microbial life-history strategies (3, 32). Coupled with measurements of total microbial abundance and the abundances of functional genes by quantitative PCR, these methods offer a comprehensive perspective on the potential strategies of microbes in response to changes in land use. From an ecological perspective, the finding that the *rrn* copy number is 20% higher in agricultural soils compared to pristine soils suggests a shift toward copiotrophic (fast-growing, resource-opportunistic) microbes in agricultural ecosystems (Fig. 1). A higher proportion of copiotrophic microorganisms may lead to a reduction in overall microbial diversity, as these organisms can dominate and outcompete slow-growing, resource-efficient oligotrophic species, which are better adapted to low-nutrient conditions like those in pristine soils (5). Copiotrophs are often more active in decomposition and nutrient cycling. This can lead to faster mineralization rates, potentially leading to increased nutrient availability in the short term and reduced soil organic matter stocks over time. While copiotrophs excel in resource-abundant conditions and are less resilient to environmental stressors (e.g., drought, salinity) (3), a 20% increase in the *rrn* copy number could reduce the stability of microbial communities in agricultural soils compared to more balanced pristine ecosystems.

Microbial life-history strategy is associated with microbial genomic and functional traits (51, 53). The abundances of *Bacteroidetes*, which had more copiotrophic groups inside, increased, and the abundance of *Acidobacteria*, which had more oligotrophic groups in cropland soils, decreased (54). The oligotrophs often find alternative ways to obtain energy for their survival by degrading complex chemicals and other environmental toxins. This is consistent with the higher abundance of the genes for xenobiotic biodegradation and metabolisms in pristine soils (Table 1). Copiotrophs, however, have a poor affinity for their substrates and mainly grow vigorously in nutrient-rich environments. Thus, we observed a higher abundance of genes responsible for transport and catabolism (Table 1), which are crucial for microbial reproduction (55) and nutrient transportation (56). While consistent changes in microbial life-history strategies and functional attributes (e.g., amino acid metabolism, xenobiotic degradation/metabolism, protein folding/sorting/degradation, as shown in Table 1) between cropland and pristine soils were identified, these findings primarily illustrate how microorganisms respond to land-use changes by adjusting their metabolic pathways. However, whether these functional categories are directly linked to microbial copiotrophic or oligotrophic strategies remains unclear. Other microbial life-history strategies might be at play, such as their increased resistance to environmental stress. The GC content did not increase in pristine soils, but microbial communities had a lower variance in genomic GC content (Fig. S4b; Fig. 2b). This indicates that pristine soils, where nutrients are limited, may select microorganisms with similar genomic traits. The codon usage bias was at the same level in cropland and pristine soils (Fig. S4d). This is probably because conserving energy can result in codon usage bias when resources are limited (57), even though codon usage bias is usually associated with a higher predicted maximum growth rate (6). Overall, the microorganisms in cropland soils are more copiotrophic (i.e., the r-strategy) than pristine soils.

Lower gene abundances of carbon hydrolysis, inorganic N transformation, and P solubilization associated with cropland environment were observed (Fig. 3). Moreover, the abundances of C, N, and P genes involved in the typical pathways decreased with the increasing *rrn* copy number (Fig. 3e). These indicate that copiotrophs in cropland soils presumably have low potentials involved in C, N, and P metabolisms. In detail, the higher relative abundances of genes associated with carbohydrate metabolisms (e.g., *chiA* and *pgu* for chitin and pectin, respectively, and *abfA*, *xylA*, *manB*, and *amyX* for hemicellulose and starch) were observed in the pristine soils (Fig. 3b through d), suggesting that the NP deficiency increases the microbial potential to decompose organic compounds in order to obtain nutrients (58). Here, we note that genes associated with monomers or more labile carbon sources could not be identified by the qPCR chip used in this study. As a result, the overall C cycling assessment overlooks a significant portion of non-polymeric C sources and is more reflective of polymeric C depolymerization. This raises uncertainty regarding whether genes involved in labile C degradation are also less essential in cropland soils compared to those for polymeric C degradation. The increased abundances of genes responsible for organic N mineralization and *nasA* and *napA* genes responsible for dissimilatory/assimilatory nitrate reduction in pristine soils (Fig. 3c) suggested that the oligotrophs may contribute to the alleviation of N deficiency by generating more NH_4_^+^ that is less leached compared to NO_3_^−^ (59, 60). This was attributed to accelerated denitrification activities caused by the excessive NO_3_^−^ input or NO_3_^−^ production from added urea (61, 62). As regards P cycling, the abundances of genes involved in phosphonate degradation, phosphate solubilization, and organophosphorus mineralization were higher in pristine than cropland soils (Fig. 3d). This suggests that microorganisms in pristine soils efficiently release available P from the fixed P pools for their metabolisms (63–65). The higher potential of microbial P solubilization in pristine soils contributes to the alleviation of P deficiency. Overall, the increased number of diverse microbial processes in pristine soils suggests that metabolic multifunctionality is an essential feature of oligotrophic microbial communities (66). This metabolic diversity allows oligotrophs to survive in resource-scarce environments as an important adaptive strategy. Further research involving measurements of microbial metabolic rates is recommended to validate whether the higher gene abundances in oligotrophs from pristine soils correlate with increased protein synthesis.

Exploring the key drivers of soil microbial life strategies provides new insights into the impact of human interventions and soil management compared to pristine factors, such as climate. Our large-scale survey suggests that climate does not contribute greatly to the microbial life-history strategies, although they greatly influence microbial diversity and community composition (67–69). This was confirmed by similar levels in the average *rrn* copy number between four climate zones, except the middle subtropical zone (Fig. S2). The SEM also revealed the small contributions of climate to the *rrn* copy number (Fig. 4b).

In general, the community-level *rrn* copy number increases with increasing soil available organic C (33). Organic matter inputs provide energy sources for copiotrophs, promote their growth, and finally increase the community-level *rrn* copy number. For example, the accumulation of litters and root exudates in pristine soils is a carbon source stimulating copiotrophic growth (70). By contrast, we demonstrated that the pristine soils with high total C had a relatively low *rrn* copy number compared to cropland soils (Fig. 1g). Consequently, the higher organic C content may not directly induce the growth of copiotrophs, as some organic matter pools in pristine soils are probably persistent. For example, in grassland topsoil, more than 50% of organic C is present in mineral-associated organic matter (71), which binds tightly to minerals or is enclosed in small microaggregates (<50 µm), making it inefficient to decompose by microorganisms (72, 73). Thus, the difference in soil organic C may not greatly explain the change in microbial life-history strategies between cropland and pristine soils. Strong evidence demonstrates that forest and grassland always suffer from N and P deficiency due to the considerable uptake of N and P by vegetation (24, 74), removal of aboveground biomass by grazing (25), as well as leaching (75). The decomposition of organic matter is generally limited if the CNP stoichiometry is not balanced, and the N and P nutrients are not supplied (22).

Cropland soils annually received a large number of mineral fertilizers, resulting in higher contents of available P and N and the high ratios of AP/TP, AN/TN, and DON/TN in cropland soils (Fig. 1d and e). N and P from fertilization are not only directly immobilized into microbial biomass but also adjust the CNP stoichiometry, accelerating the decomposition of organic matter, leading to the supply of more available organic C for copiotrophic growth. Most cropland soils also receive organic materials (e.g., manure, straw, or compost) (76, 77), which are less chemically persistent with forest and grassland residues. The decomposition of available organic C, together with N and P supplies, also contributed to the *rrn* copy number increase. Although the precipitation and temperature in cropland soils were coincidentally higher than those in pristine soils (Fig. 1b), these climatic factors only contributed to microbial diversity and had no significant effects on microbial strategies (Fig. 4). Other human disturbances, such as plowing and irrigation, in addition to mineral fertilizer inputs, alter the soil physical structure and affect the microbial community (20, 21). The similar levels of soil water content and texture (except silt content) between cropland and pristine soils (Fig. 1c) indicated that these parameters explained little about the differences in microbial life-history strategies. The accumulation of organic matter in pristine soils decreases the bulk density, providing more oxygen availability for microbial growth. The SEM model still indicated that the bulk density did not contribute largely to the microbial life-history strategies (Fig. 4a). Rather, the N and P contents dominantly explained the total variance of the community-level *rrn* copy number rather than soil physical properties (Fig. 4a). This pattern was further verified by four agroecosystems subjected to long-term NP fertilization. The elevated average *rrn* copy numbers observed in soils treated with NP fertilizers compared to those without underscored the significant influence of N and P contents in favoring candidate r-strategists in croplands (Fig. 5). Consequently, the nutrient input was a dominant factor in creating a more copiotrophic environment for microbial growth in cropland soils, while pristine soils without nutrient inputs represent an oligotrophic environment.

### Conclusions

**Fig 6:**
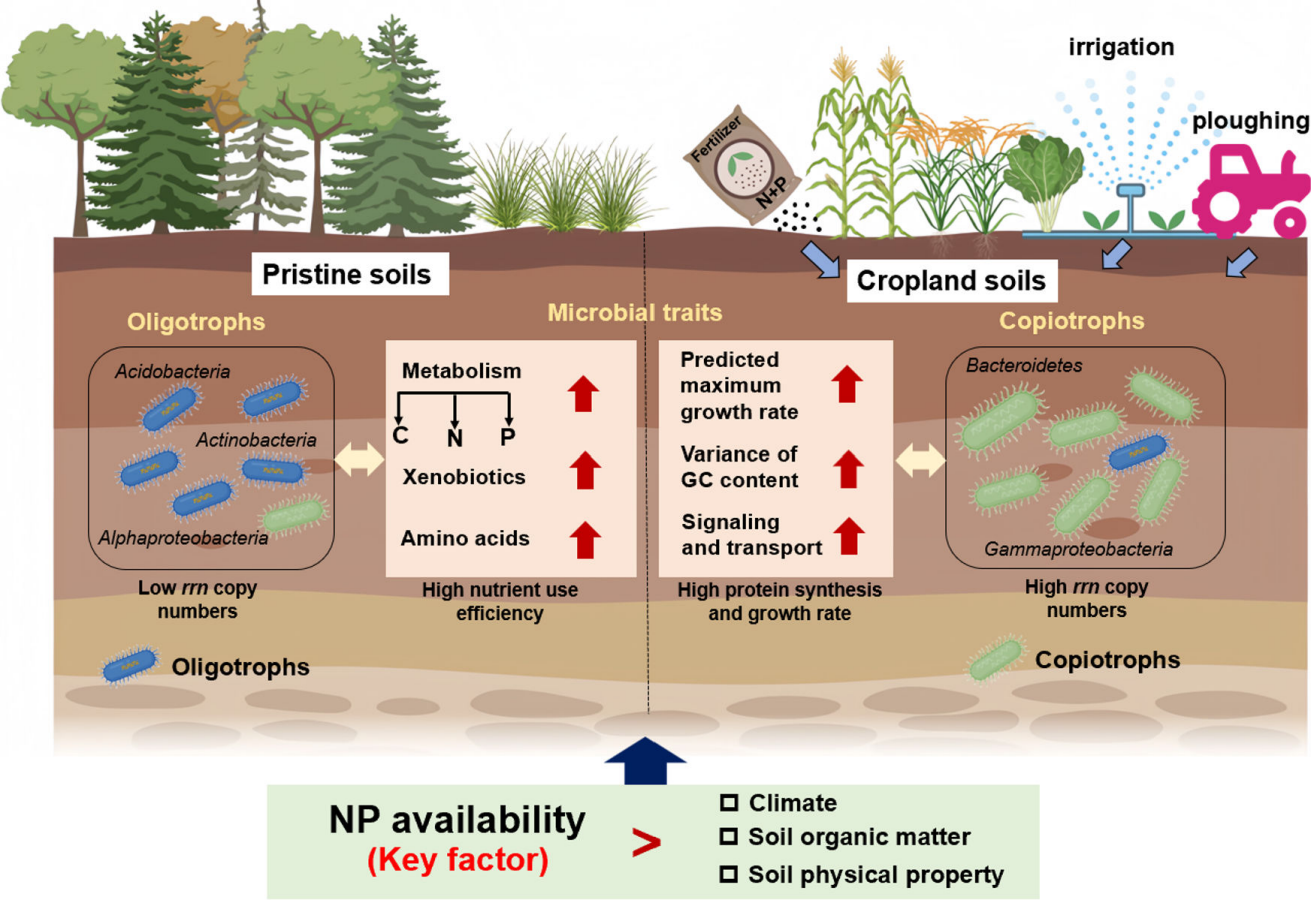
Conceptual diagram illustrating the differences in microbial life-history strategies between cropland and pristine soils and the associated dominant contributors. The application of nitrogen and phosphorus mineral fertilizers creates a nutrient-rich environment in cropland soils. This leads to an increase in copiotrophs in cropland soils, revealed by the *rrn* copy numbers and genomic traits, whereas in pristine soils with low contents of N and P, microbial communities have more K-strategists, as revealed by the abundant genes for C, N, P, and amino acid metabolism to efficiently acquire nutrients.

Our large-scale survey revealed that land use types lead to contrasting microbial life-history strategies and functions in soil (Table S2). The microorganisms in cropland soils tend to be more copiotrophic, whereas those in pristine soils are more oligotrophic (Fig. 6). By going beyond localized experiments, large-scale surveys provided a broader, more realistic context and enhanced the external validity of the findings. Then, we employed controlled field experiments to verify that the high available N and P contents caused by intensive fertilization in croplands were the main driver for the development of microbial r-strategists. Although we evaluated microbial strategies by employing molecular techniques with metagenomics data, without direct measurements of microbial biological processes, the predictive approaches still offer a comprehensive view of potential microbial responses to different land uses, providing a crucial framework for understanding microbial ecological patterns across wide gradients of environmental conditions.

## SUPPLEMENTAL MATERIAL

Supplemental material msystems.00178-25-s0001.docx Fig. S1 to S4; Tables S1 to S3.

ASM does not own the copyrights to Supplemental Material that may be linked to, or accessed through, an article. The authors have granted ASM a non-exclusive, world-wide license to publish the Supplemental Material files. Please contact the corresponding author directly for reuse.
