# Supplementary material for: Microbial life-history strategies and genomic traits between pristine and cropland soils
Source: mSystems. 2025 Apr 16;10(5):e00178-25. doi: 10.1128/msystems.00178-25 (PMC12090741; doi:10.1128/msystems.00178-25)
Supplement: Supplemental material — Fig. S1 to S4; Tables S1 to S3. [file msystems.00178-25-s0001.docx]

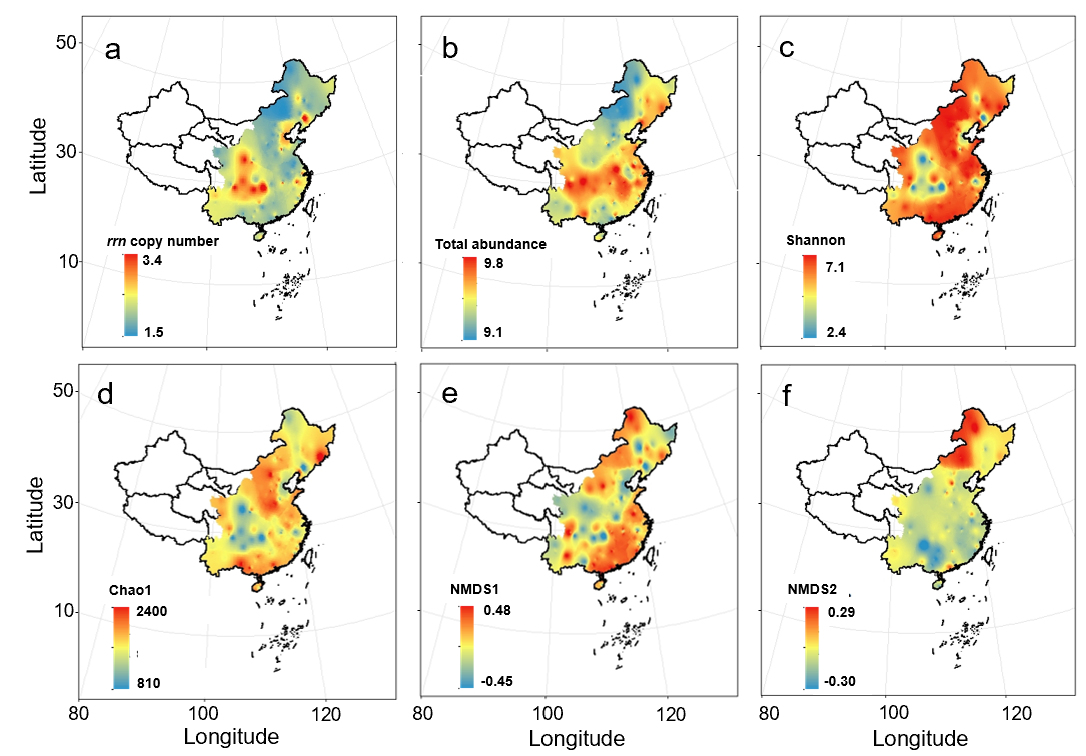


Fig. S1. Distribution of microbial diversity and life-history strategies across sampling regions. Maps of bacterial average *rrn* copy number (a), total abundance (by log10 transformation) (b), alpha diversity (Shannon and Chao 1) (c and d) and beta diversity (NMDS1 and NMDS2) (e and f) across sampling regions using kriging interpolation.


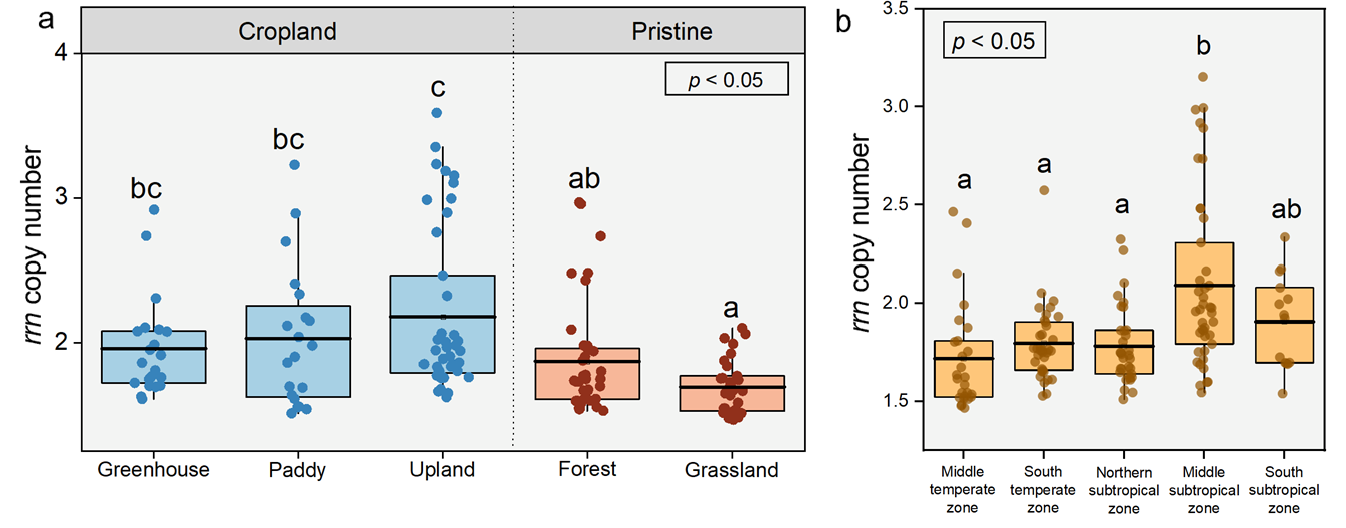


Fig. S2. Microbial average *rrn* copy number in five land uses: upland, paddy, greenhouse, forest and grassland (a). And the microbial average *rrn* copy number at the community level between five climate zones (b). Box plots indicate the means (horizontal lines), 1st and 3rd quartiles (boxes), and 1.5× interquartile range (whiskers).


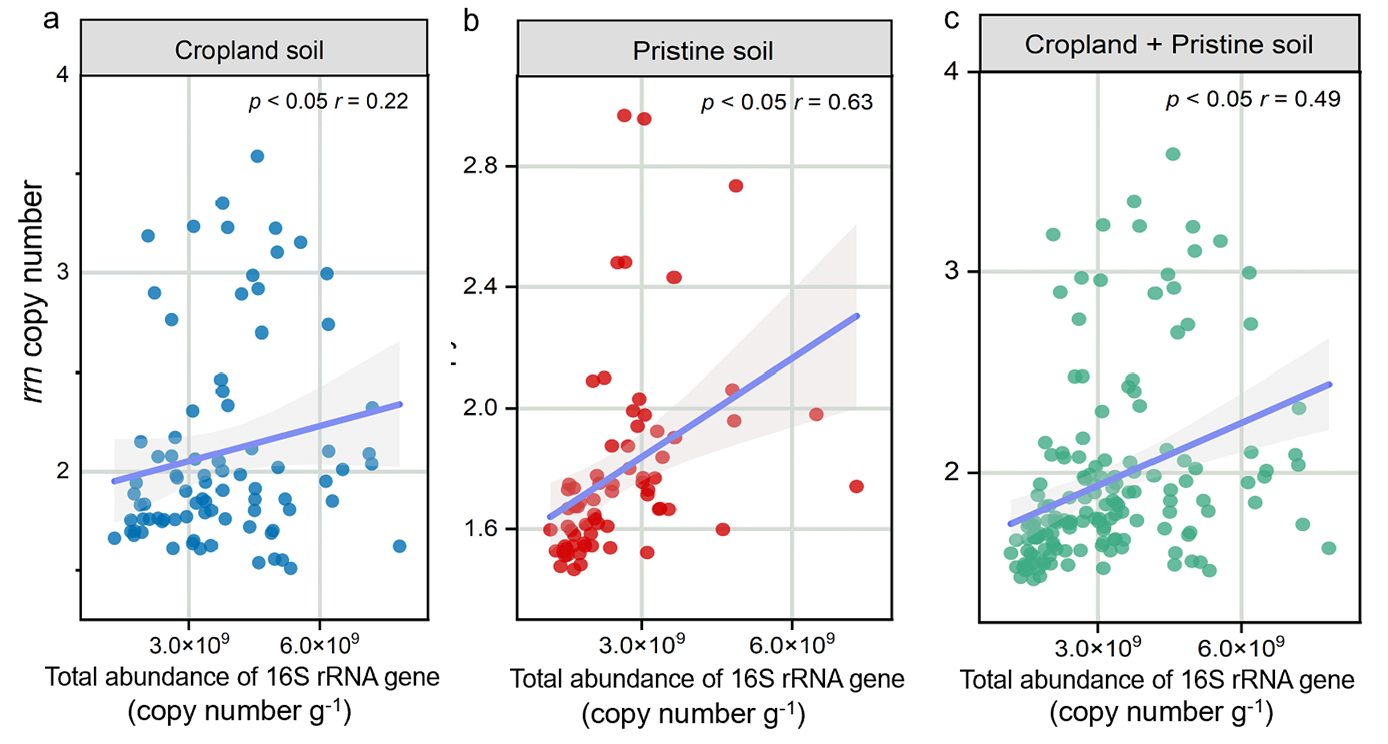


Fig. S3. Spearman’s correlations between the average *rrn* copy number and Total abundance of 16S rRNA gene across cropland soils (a), pristine soils (b) as well as cropland and pristine soils (c).


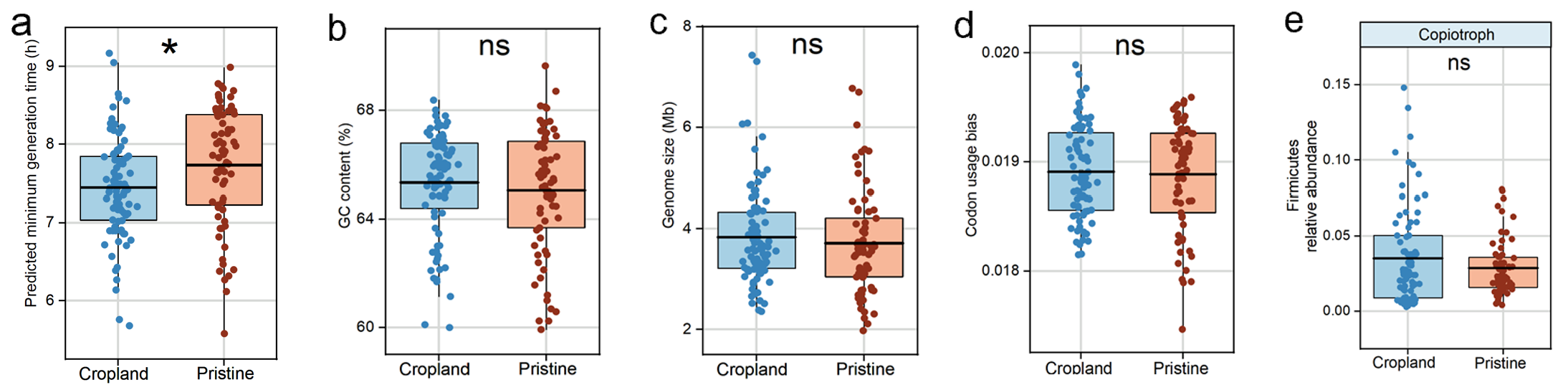


Fig. S4. Predicted minimum generation time (a), GC content (b), genome size (c), codon usage bias (d), and *Firmicutes* (e) between cropland and pristine soils. Box plots indicate the means (horizontal lines), 1st and 3rd quartiles (boxes), and 1.5× interquartile range (whiskers). The differences between cropland and pristine soils were performed by the linear mixed-effects models. **p* < 0.05, ns not significant.

Table S1. Ecological attributes that correspond to copiotrophic and oligotrophic groups of bacteria.

| traits | Copiotrophs | Oligotrophs |
| --- | --- | --- |
| Growth rate | High maximum growth rate in resource-rich environments. ^1^ | Low maximum growth rate, disadvantaged in copiotrophic environments. ^1^ |
| Substrate affinity | Low specific affinity for substrates, less competitive for substrate when resources are limited. ^2^ | High affinity, excellent competitors  in oligotrophic environments. ^2^ |
| Carbon use efficiency | Low. ^3^ | High, efficient growth is an adaptation of bacteria with few *rrn* copies. ^3^ |
| Tolerance to environmental stress | High sensitivity to environmental stress. ^1^ | Maintaining viability under environmental stress. ^1^ |
| rRNA operon copy number | High. ^4^ | Low. ^4^ |
| GC content | Low. ^5^ | High, a higher genomic GC content can enhance the thermotolerance of a microorganism. ^5^ |
| Variance of GC content | High. ^5^ | Low, the stressful environment enriched microbes with similar GC contents and limited their variability. ^5^ |
| Translational power | High, the translational power is three-to  fourfold higher among bacteria that  respond rapidly to nutrient availability than among bacteria that respond slowly. ^3^ | Low. ^3^ |

Superscript numbers refer to sources: 1, Fierer (2007); 2, Button (1993); 3, Roller (2016); 4, Klappenbach et al. (2000); 5, Chen et al. (2021);

Table S2. Comparison of life history strategies of microorganisms in cropland (r-strategy) and pristine soils (K-strategy).

| traits | r-strategy | K-strategy |
| --- | --- | --- |
| Predicted growth rate (h^-1^) | High maximum growth rate in cropland soils | Low maximum growth rate, advantaged in pristine soils |
| Predicted minimum generation (h) | Low | High |
| The average *rrn* copy number | High | Low |
| Total abundance of 16S rRNA gene (copy number g^-1^) | High | Low |
| GC content (%) | Same level | Same level |
| Variance of GC content | High | Low, the stressful environment enriched microbes with similar GC contents and limited their variability |
| Genome size (Mb) | Same level | Same level |
| C metabolism genes | Low | High, to efficiently acquire nutrients |
| N cycling genes | Low | High, to efficiently acquire nutrients |
| P cycling genes | Low | High, to efficiently acquire nutrients |
| The abundance of *Acidobacteria* | Low | High |
| The abundance of *Actinobacteria* | Low | High |
| The abundance of *Bacteriodetes* | High | Low |

Table S3. Annotation of specific genes involved in C, N and P metabolisms quantified by high-throughput qPCR-based chip.

| C cycling | | N cycling | | P cycling | |
| --- | --- | --- | --- | --- | --- |
| C degradation | *amyX* | N cycling | *amoB* | P cycling | *phoD* |
| C degradation | *apu* | N cycling | *napA* | P cycling | *ppx* |
| C degradation | *iso-plu* | N cycling | *nasA* | P cycling | *cphy* |
| C degradation | *pox* | N cycling | *nirK1* | P cycling | *bpp* |
| C degradation | *abfA* | N cycling | *nirK3* | P cycling | *phoX* |
| C degradation | *lig* | N cycling | *gdhA* | P cycling | *ppk* |
| C degradation | *cex* | N cycling | *hao* | P cycling | *pqqC* |
| C degradation | *pgu* | N cycling | *hzsA* | P cycling | *gcd* |
| C degradation | *amyA* | N cycling | *amoA1* | P cycling | *phnK* |
| C degradation | *cdh* | N cycling | *nirS2* |  |  |
| C degradation | *glx* | N cycling | *nosZ1* |  |  |
| C degradation | *manB* | N cycling | *nxrA* |  |  |
| C degradation | *mnp* | N cycling | *ureC* |  |  |
| C degradation | *naglu* | N cycling | *narG* |  |  |
| C degradation | *sga* | N cycling | *nifH* |  |  |
| C degradation | *chiA* | N cycling | *nirK2* |  |  |
| C degradation | *exo-chi* | N cycling | *nirS1* |  |  |
| C degradation | *xylA* | N cycling | *hzo* |  |  |
| C fixation | *cdaR* | N cycling | *hzsB* |  |  |
| C fixation | *smtA* | N cycling | *amoA2* |  |  |
| C fixation | *aclB* | N cycling | *nirS3* |  |  |
| C fixation | *acsB* | N cycling | *nosZ2* |  |  |
| C fixation | *mct* |  |  |  |  |
| C fixation | *rbcL* |  |  |  |  |
| C fixation | *frdA* |  |  |  |  |
| C fixation | *korA* |  |  |  |  |
| C fixation | *accA* |  |  |  |  |
| C fixation | *acsA* |  |  |  |  |
| C fixation | *acsE* |  |  |  |  |
| C fixation | *mcrA* |  |  |  |  |
| C fixation | *pccA* |  |  |  |  |
| Methane metabolism | *pqq-mdh* |  |  |  |  |
| Methane metabolism | *mmoX* |  |  |  |  |
| Methane metabolism | *mxaF* |  |  |  |  |
| Methane metabolism | *pmoA* |  |  |  |  |

**References**

Button DK. 1993. Nutrient-limited microbial growth kinetics: overview and recent advances. *Antonie van Leeuwenhoek* 63:225-235.

Chen Y, Neilson JW, Kushwaha P, Maier RM, Barberan A. 2021. Life-history strategies of soil microbial communities in an arid ecosystem. *The ISME Journal* 15:649-657.

Fierer N, Bradford MA, Jackson RB. 2007. Toward an ecological classification of soil bacteria. *Ecology* 88:1354-1364.

Klappenbach JA, Dunbar JM, Schmidt TM. 2000. rRNA operon copy number reflects ecological strategies of bacteria. *Applied and Environmental Microbiology* 66:1328-1333.

Roller BRK, Stoddard SF, Schmidt TM. 2016. Exploiting rRNA operon copy number to investigate bacterial reproductive strategies. *Nature Microbiology* 1:16160.
